# Applicability of machine learning technique in the screening of patients with mild traumatic brain injury

**DOI:** 10.1371/journal.pone.0290721

**Published:** 2023-08-24

**Authors:** Miriam Leiko Terabe, Miyoko Massago, Pedro Henrique Iora, Thiago Augusto Hernandes Rocha, João Vitor Perez de Souza, Lily Huo, Mamoru Massago, Dalton Makoto Senda, Elisabete Mitiko Kobayashi, João Ricardo Vissoci, Catherine Ann Staton, Luciano de Andrade

**Affiliations:** 1 Postgraduate Program in Management, Technology and Innovation in Urgency and Emergency, State University of Maringa, Maringa, Parana, Brazil; 2 Postgraduate Program in Health Sciences, State University of Maringa, Maringa, Parana, Brazil; 3 Department of Medicine, State University of Maringa, Maringa, Parana, Brazil; 4 Duke Global Health Institute, Duke University Medical Center, Durham, North Carolina, United States of America; 5 Postgraduate Program in Biosciences and Physiopathology, State University of Maringa, Maringa, Parana, Brazil; 6 Postgraduate Program in Computer Sciences, State University of Maringa, Maringa, Parana, Brazil; Staffordshire University, UNITED KINGDOM

## Abstract

Even though the demand of head computed tomography (CT) in patients with mild traumatic brain injury (TBI) has progressively increased worldwide, only a small number of individuals have intracranial lesions that require neurosurgical intervention. As such, this study aims to evaluate the applicability of a machine learning (ML) technique in the screening of patients with mild TBI in the Regional University Hospital of Maringá, Paraná state, Brazil. This is an observational, descriptive, cross-sectional, and retrospective study using ML technique to develop a protocol that predicts which patients with an initial diagnosis of mild TBI should be recommended for a head CT. Among the tested models, he linear extreme gradient boosting was the best algorithm, with the highest sensitivity (0.70 ± 0.06). Our predictive model can assist in the screening of mild TBI patients, assisting health professionals to manage the resource utilization, and improve the quality and safety of patient care.

## Introduction

The increase in the demand even in mild cases of illness associated with low capacity of health care services leads to overcrowding of emergency health services in different countries of the world [1, 2]. Among the diseases with low severity which is on expansion, there is the mild traumatic brain injury (TBI), which corresponds to 81.01% of all registered 69 million cases annually or approximately 740 cases per 100,000 people worldwide [3, 4]. Previous studies have shown the overuse of the head computed tomography (CT) in patients with suspected mild TBI. [5, 6]. However, only 5.2 to 9.4% of patients have intracranial lesions and 0.2 to 3.5% require neurosurgical intervention [7–9].

An example of this overcrowding can be seen in Brazil, where the probability of hospitalization due to TBI has tripled between 2001 and 2017 [10]. In 2019 alone, the country witnessed over 100,000 cases of TBI-related hospitalizations, resulting in an estimated cost of 18,489,452.36 Brazilian reals (equivalent to U$4,587,953.44). On average, each patient required approximately 6.3 days of hospitalizations [10].

Early recognition of high-risk clinical factors can help identify a subset of patients who are likely to have intracranial lesions and increase the survival of individuals [11–13]. However, performing CT in all patients is not feasible, and time and resources should be focused on those most likely to have lesions that require neurosurgical intervention [14].

To solve this problem several studies have been carried out, and guidelines have been developed and validated bringing a set of prediction rules to help in making appropriate decisions to determine which patients with mild TBI are indicated to undergo CT of the head. Among them, the most cited are the Canadian CT Head Rule and New Orleans Criteria [15, 16].

Although these guidelines aid in screening patients with mild TBI in their countries of origin, the application of these guidelines in Brazil leads to external validity issues, which is limited to patients who had loss of consciousness, post-traumatic amnesia or witnessed disorientation [8, 11, 17–20]. Furthermore, these guidelines performed differently in countries with distinct socioeconomic situations, due to the difference in the target audience profile [9], mechanism of trauma [21, 22] and availability of financial resources [8, 23].

These gaps demonstrate that protocols should be validated and applied according to the intrinsic characteristics of the target audience, including culture, sociodemographic patterns, epidemiological profile, and the availability of medical resources and equipment in each location [11, 17]. Thus, finding and improving the most contextually relevant and location-specific clinical decision protocol has been the aim of study of Fournier et al., which adjusted the Canadian CT head rule to people 75 years old or over [24]. Vedin et al., in their turns, studied the applicability history of patients in the final decision of the people under 59 years old [13].

Previous research has shown that machine learning techniques, specifically grouping and analyzing variables that indicate an inclination to certain pathology, can provide a global view of the patients’ clinical status [25, 26]. This global view can then assist in complex clinical decision making, and reduce patients’ time spent in health centers by automating several functions [27]. In addition, machine learning models have been shown useful in predicting hypertension in Qatar [25], diabetes in Brazil [28] and TBI in Tanzania [26].

Despite these advancements, there are still no studies focused on the development of risk predictor tools for intracranial injuries in adults with mild TBI in Brazil. Hence, this study aims to evaluate the applicability of machine learning to screen patients with mild TBI evaluated at the Regional University Hospital of Maringa using a supervised classification model.

## Methodology

### Study design

This is an observational, descriptive, cross-sectional, and retrospective study, using machine learning (ML) developed computational models to predict which patients with an initial diagnosis of mild TBI are recommended to undergo head CT. We evaluated the need for imaging as lesions identified on CT which required evaluation by neurosurgeons and presented the following outcomes: requiring hospitalization for neurological observation or neurosurgical intervention, requiring transfer to advanced neurosurgical care, and patients who died with TBI as the main cause.

### Data sources

This project was approved by the Ethics Committee on Research Involving Humans at the State University of Maringá (Registration No. 3.952.659/20). We used secondary data from electronic medical records of patients with mild TBI evaluated at the Regional University Hospital of Maringá (HUM) in 2018 available in the Hospital and Outpatient Management System of the Unified Health System. For this reason, the resolution number 466/2016 of the Brazilian Ministry of Health allows us to work with this data without an informed consent form.

The Regional University Hospital of Maringa (HUM) is one of the referral hospitals in the Northwest macroregion of the state of Paraná and is responsible for the care of individuals with complex traumatic injuries. Patients suffering injuries who are suspected or confirmed to have TBI are attended first by surgical clinic physicians who decide to do a CT and requests neurosurgeon evaluation when it is necessary. The HUM is equipped with, among other equipment, a computerized tomography (Lightning Aquilion 80-row multi-detector—Canon Medical System), which works with 80-slices, 24 hours a day seven days per week.

### Data selection

#### Patients’ selection

In compliance with data protection regulations, for safeguarding individuals’ personal data, the hospital provided the data after strictly followed after strictly following the principles about the principles regarding the treatment and processing of data for this study. Data anonymization techniques were employed to protect privacy and confidentiality.

The initial dataset was composed of data of 2,360 patients 14 years old or older with TBI who presented at the HUM emergency Department, between January 1^st^ and December 31^st^ of 2018. Of all these reports, 96.91% or 2,287 patients had Glasgow Coma Scale (GCS) scores between 13 and 15.

Previous literature cites people over 65 years old [29] and/or with GCS scores equal or less than 13 have an increased risk of being diagnosed with intracranial lesions [30]. As this information can induce the predictive model to provide a positive output, it is recommended to perform head CT for such individuals regardless of other clinical conditions [29, 30]. Furthermore, to avoid the possibility of duplicated data, when a patient presents at the HUM Emergency Department less than one week after their prior visit with the same mechanism of trauma, the second visit was excluded from the dataset, resulting in 1851 remaining observations.

#### Variables’ selection

Initially, twenty-eight variables were available; we excluded time of attendance, variables with more than 25% missing data, as well as those that generated doubts in the interpretation, to avoid underestimation of the data. Other variables such as change in cognitive responses (ie, pupil dilatation, neurological deficit and loss of consciousness) since there was almost none with visual or neurological alteration during clinical analysis were also excluded.

To mitigate overfitting, lesions suggestive of a potential brain injury, including those confirmed by imaging tests like Skull x-ray and cranial tomography performance were excluded. Additionally, elements displaying a Pearson correlation of over 70% (38) were also removed from the data.

Hence, after evaluating the reliability of the variables and their association with the occurrence of intracranial lesions, only 10 variables, which were not directly correlated among them, were selected for machine learning prediction model development (Box 1).

**Box 1. Variables selected for this study**

*The following parameters were considered as high impact trauma: traffic crashes involving pedestrians, ejection of the driver and/or passenger from the vehicle in movement, vehicle speed above 60 km/h, physical aggression with a blunt object, and fall from at least one meter or five steps, according to Canadian protocol [18]*.

**For the evaluation of post-traumatic amnesia, loss of consciousness, and dizziness, the signs and symptoms reported by the patient were analyzed along with those described by third parties and/or observed during the medical examination.

### Outcome

We evaluated the performance of six machine learning models to correctly identify individuals with head CT findings among patients with mild TBI attended by the HUM ED based on physiological characteristics as predictor variables.

### Statistical test

The chi-square statistical test through the software RStudio version 4.1.0 [31] was used for comparisons between patient data with and without head CT finding, adopting a significance level of 5% (p≤0.05).

### Development of prediction models and predictors

The TRIPOD (Transparent reporting of a multivariable prediction model for individual prognosis or diagnosis) protocol [32] was adopted to standardize the development of supervised and classificatory machine learning algorithms. The package caret [33] was used to train and create the models.

**Fig 1:**
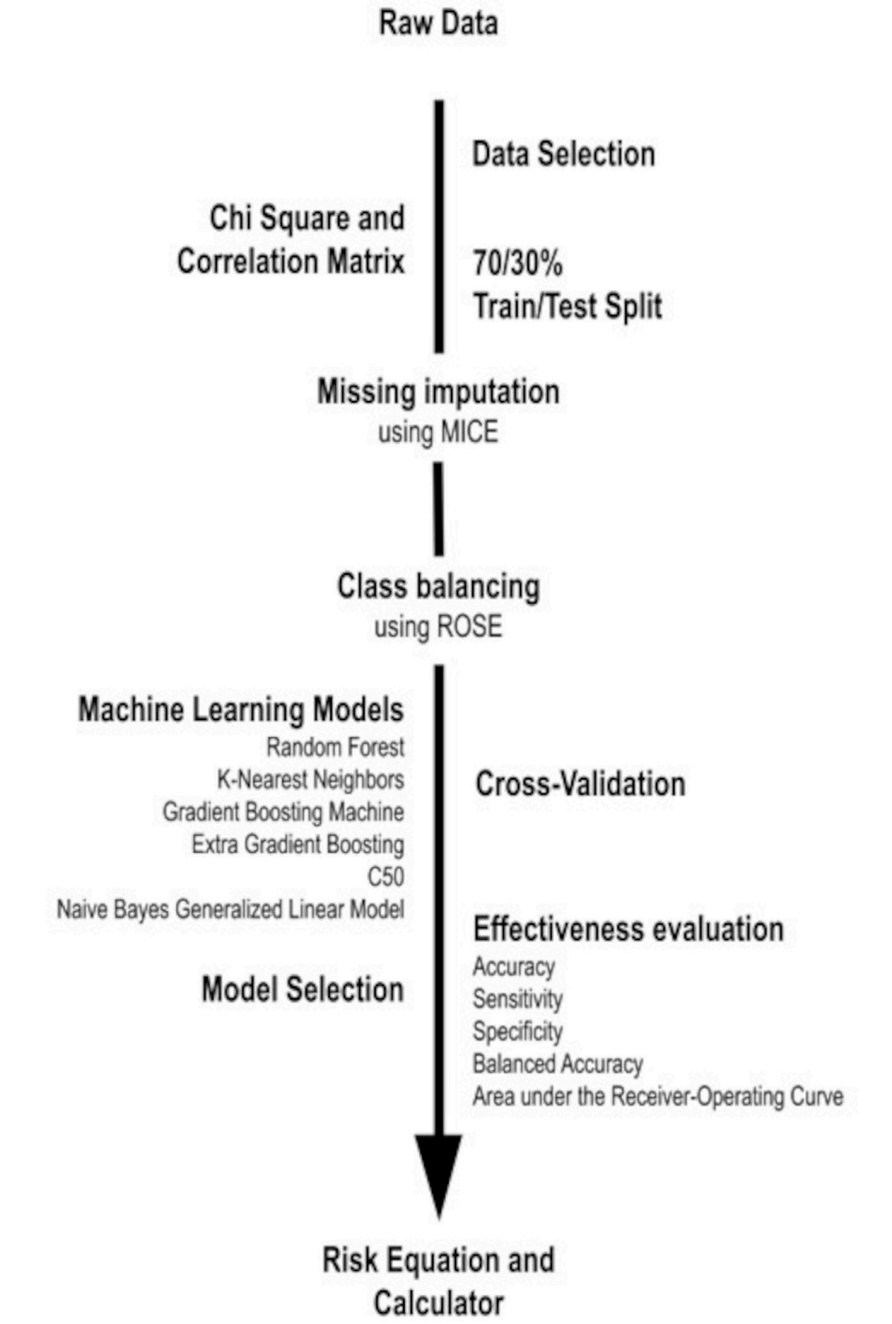
Predictor model development process.

The model development process was as follows: data selection by exclusion and inclusion criteria, imputation of missing data and cross-validation, testing of six computational algorithms to assess their effectiveness, and selection of the best model based on sensitivity. Based on the performances of the algorithms, a prototype of a risk equation to predict changes in head CT, called predict CT-Calculator, was built using the best models (Fig 1).

The data selected in this research was divided into a 70/30 (train/test) ratio and the missing values were imputed 10 times using the package MICE (multivariate imputation by chained equations) [34] for 3 tree-based algorithms (Random Forest, Gradient boosting machine and C5.0) and 3 non-tree-based algorithms (K-nearest neighbors, Linear extreme gradient boosting and Naive bayes generalized linear models), using caret package [33] as described below.

1. *Random forest* (RF): a combination of decision trees that makes the analysis more complex, increasing the efficiency of prediction [35, 36];
2. *K-Nearest Neighbors* (KNN): a method used to classify a given parameter based on the results obtained from its closest neighbors, that is, it uses likelihood to classify data [37, 38].
3. *Gradient boosting machine* (GBM): a strong model that combines several weak models (e.g., decision tree), optimizing the predictions through a boost gradient [39].
4. *Linear extreme gradient boosting (XGB)*: a linear highly efficient gradient boosting model [40].
5. *C5*.*0*: a model based on a decision tree or collection of rules. It preserves factors and other classes, prevents the automatic creation of false variables, and facilitates their implementation and understanding compared to other models, such as Supporting Vector Machine and neural networks [41, 42].
6. *Naive Bayes generalized linear models* (NBglm): a classificatory Bayesian model based on the occurrence of a given event for data with a normal distribution [43, 44].

Since more than 90% of patients did not have a finding on imaging, the proportion of negative and positive imaging results were corrected using the package Random Over-Sampling Examples (ROSE) to lead with binary class imbalance [45]. The cross-validations were performed 100 times. The test and train datasets were divided in 10 parts/each (for each of 10 validations, nine parts were used to train and one for internal validation) and this process was repeated 10 times, according to cross-validation methodology described by Bergamini et al. [46], adapted by the authors.

We chose the performance metrics that would show the accuracy of the model in predicting a binary classifier of mild brain injury. We chose the standard general performance metrics of accuracy (ACCU), sensitivity (SENS), specificity (SPEC), and area under the receiver operating characteristic curve (AUROCC) [28, 47–51].

We also report the balanced accuracy (BALACCU) as a measure of a classification performance that takes into account both positive and negative. It is calculated as (SENS + SPEC) / 2. Balanced accuracy can be useful when the class distribution is imbalanced, as it considers both classes equally [28, 47–51]. Finally, we decided to report the probability of false negatives (PFN) because we wanted to know the probability of misclassification of a positive instance as negative [25, 28, 47–51], taking in account the variation of results for each imputation.

Based on sensitivity, the probability of the true positive results among all positive samples, we chose the best model and plotted its mean receiver operating characteristic curve for each imputation using the package pROC [52]. The algorithm with highest sensitivity was also used to develop the prototype of the risk equation [26], to help set the priority of CT scans. This algorithm was built into a website where health professionals can input patient data. The software then calculates the probability of the individuals to have head CT findings, as described in the discussion of this paper.

## Results

The mean age of participants was 43±20 years and 1556 (67.60%) were male. In addition, 1609 (70.35%) patients were referred to the hospital by the prehospital care service, Mobile Emergency Care Service (SAMU) or the Integrated Emergency Trauma Care Service (SIATE), 173 (7.56%) were transferred from another hospital, and 504 (22.04%) self-presented to HUM ED.

The most common mechanism of trauma was road traffic injuries (42.59%), followed by a fall from standing height (29.56%), physical aggression (14.25%), and a fall from height (8.61%). Of these, 407 (17.80%) were classified as dangerous mechanisms. In the initial evaluation of the patient with mild TBI, there was a predominance of GCS equal to 15 (86.84%), followed by GCS equal to 14 (10.10%) and GCS equal to 13 (2.17%).

It was also observed that 19.14% (438/2287) of patient encounters occurred in July and December 2018, but 20% (15/74) of the positive head CT findings occurred in May. Furthermore, although head CT was performed in 70.09% (1603/2287) of the patients, only 4.62% of them (74/1603) showed positive imaging results.

When analyzing only the patients chosen for the prediction (1851), it was observed that there were statistical differences between patients with and without changes in the head CT in the following parameters: dangerous mechanism, GCS, amnesia, dizziness, headache, vomiting and/or nausea, and convulsion (Table 1).

**Table 1:** Comparison of attendances of patients with and without changes in head computed tomography among patients with mild traumatic brain injury treated at the University Hospital of Maringa in 2018.

Parameter	Change in tomography		p-value
	**Yes**	**No**	
**High impact trauma**			0.005
Yes	0015	0340	
No	0025	1471	
**GCS**			<0.001
14	0019	0177	
15	0021	1620	
**Amnesia**			<0.001
Yes	0026	0.437	
No	0009	1029	
Not available	0000	0345	
**Loss of consciousness**			0.100
Yes	0014	0390	
No	0020	1056	
Not available	0006	0365	
**Dizziness**			0.016
Yes	0005	0068	
No	0035	1743	
**Headache**			0.003
Yes	0029	0477	
No	0019	1217	
Not available	0001	0117	
**Vomiting and/or nausea**			<0.001
Yes	0012	0188	
No	0028	1616	
Not available	0000	0007	
**Convulsion**			<0.001
Yes	0005	0014	
No	0035	1797	
**Supraclavicular lesion**			0.515
Yes	0017	0887	
No	0023	0924	

**Fig 2:**
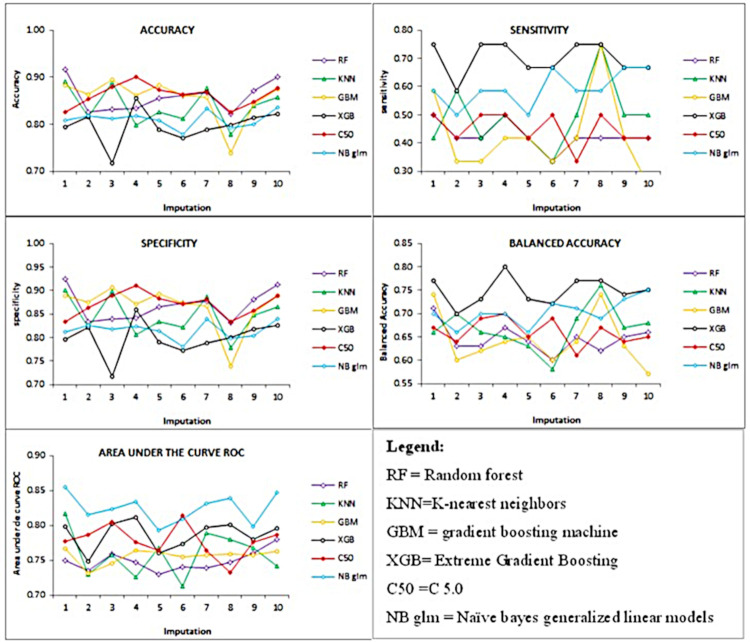
Line graphic of accuracy, sensitivity, specificity, balanced accuracy and area under the receiver operating characteristic curve for different models tested.

The mean values and standard deviation of the computational models were 0.84±0.04 for accuracy, 0.51±0.13 for sensitivity, 0.84±0.04 for specificity, 0.68±0.05 for balanced accuracy, 0.78±0.03 for AUCROC (Fig 2).

Among the tested models, the Extreme Gradient Boosting was considered the best computational model because it showed higher sensitivity (Table 2).

**Table 2:** Mean values and standard deviation of accuracy (ACCU), sensitivity (SENS), specificity (SPEC), balanced accuracy (BALACCU), and area under the receiver operating characteristic curve (AUROCC) for the different models tested.

Metric Model	ACCU	SENS	SPEC	BALACCU	AUCROC
**RF**	0.86±0.03	0.43±0.05	0.87±0.03	0.65±0.03	0.75±0.01
**KNN**	0.84±0.04	0.49±0.11	0.85±0.04	0.67±0.05	0.76±0.03
**GBM**	0.86±0.04	0.43±0.14	0.87±0.05	0.65±0.06	0.76±0.01
**C5.0**	0.86±0.02	0.45±0.06	0.87±0.02	0.66±0.03	0.78±0.02
**XGB**	0.80±0.04	0.70±0.06	0.80±0.04	0.75±0.03	0.79±0.02
**NBglm**	0.81±0.02	0.59±0.06	0.82±0.02	0.70±0.03	0.82±0.02

**Label:** RF = Random Forest, KNN = K-nearest neighbors, GBM = Gradient Boosting Machine, XGB = Linear Extra Gradient Boosting, NBglm = Naïve bayes generalized linear models.

**Fig 3:**
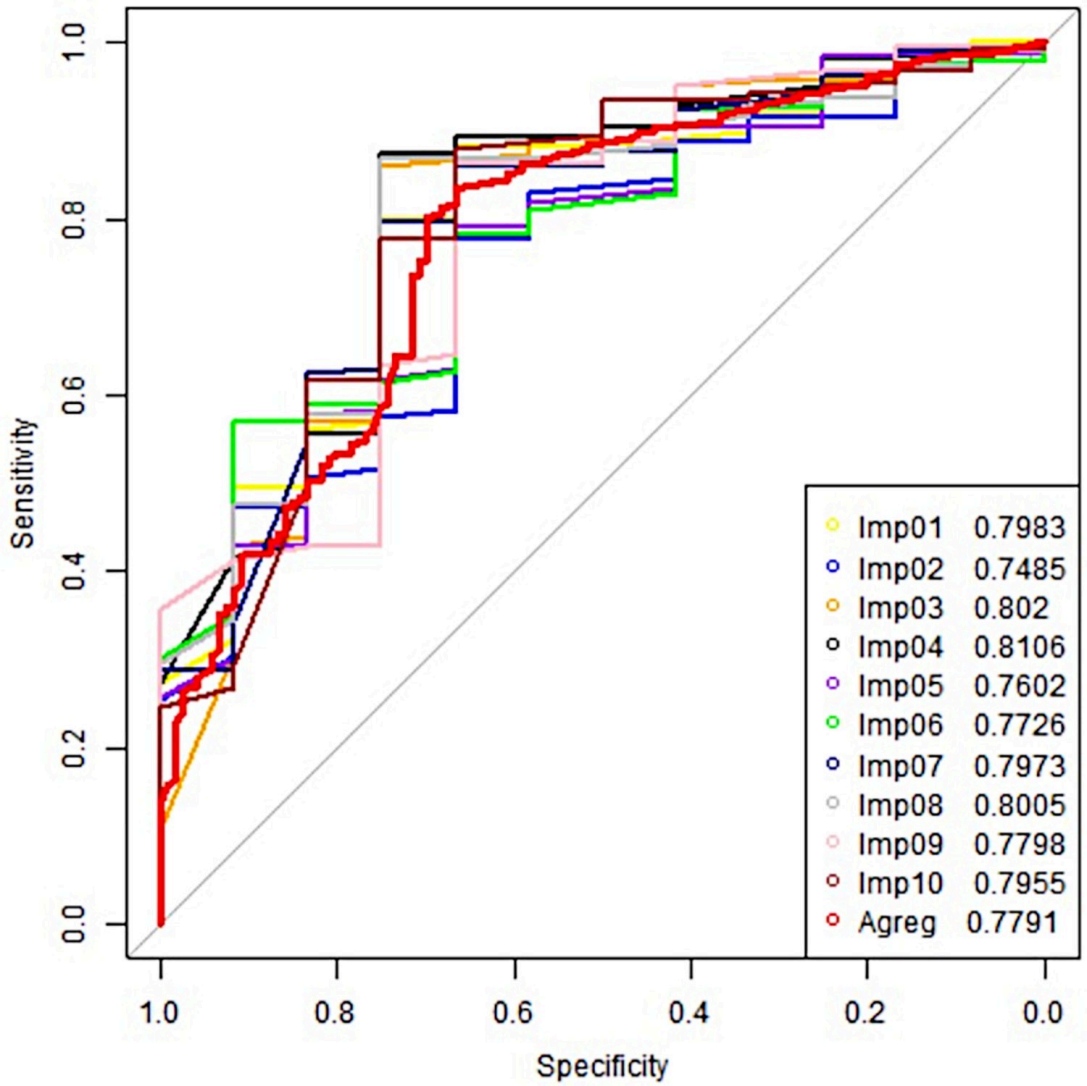
Imputation repetitions of the receiver operating characteristic curve of the best prediction model (linear extreme gradient boosting) among the parameters selected in patients with mild traumatic brain injury attended at the University Hospital of Maringá, in 2018. Imp01 = Result of first imputation; Imp02 = Result of second imputation; Imp03 = Result of third imputation; Imp04 = Result of fourth imputation; Imp05 = Result of fifth imputation; Imp06 = Result of sixth imputation; Imp07 = Result of seventh imputation; Imp08 = Result of eighth imputation; Imp09 = Result of ninth imputation; Imp10 = Result of tenth imputation; Agreg = Mean value of all imputation.

The chosen computational model (linear extreme gradient boosting) also presents a similar ROC curve profile for each data imputation (Fig 3).

## Discussion

Research focused on developing better performing prognostic methods has increased due to scientific advancements and the emerging use of machine learning (ML) algorithms. However, gaps in literature indicate that ML has not been used in screening patients with mild TBI specifically in developing countries. Hence, we aimed to fill that gap by examining the performance of six classification supervised ML algorithms.

All of the algorithms tested showed good accuracy (80 to 86%) and specificity (80 to 87%) but low sensitivity (43 to 70%). To determinate the best model, we adopted sensitivity as a reference, since it identifies the positive outcome among the samples (show the probability of true positive outcome among all the results predicted as positive) [25] and observed that XGB had the best performance agreeing with the results obtained to predict the clinical outcome of patients undergoing surgery for lumbar spinal stenosis in the Netherlands [53].

Even though the sensitivity is lower than the predictions the risk of recovery in patients with TBI in general [26] or optimizing the sensitivity with a very low specificity [54], the methodology developed in this study can help to set priority of CT scans in developing countries, but it cannot yet be used to make the final decision.

Finding the patients with higher probability of positive imaging results can aid in prioritizing attendance, reducing the patient exposure to ionizing radiation, and decreasing financial costs associated with Emergency Department examinations for traumatic brain injury [5, 29, 55]. However, some adjustments must be made to increase the sensitivity for better applicability in health centers.

In the United States of America, when the electroencephalogram was associated with the clinical symptoms of the patients, such as loss of consciousness, headache, nausea and/or vomiting, light and/or sound sensitivity, confusion and memory dysfunction, for the brain injury prediction in people affected by mild TBI, the researchers obtained an accuracy of 91% using the gradient boosting model [5] and sensitivity of 86% and specificity of 71% with the genetic algorithm model based on linear discriminant functions [56].

Since the main injury mechanism in developed countries is the fall of elderly people [21], while for low- and middle-income countries it is traffic accidents and violence involving young people [22], usually, it is not possible to use the same algorithms in the countries with different socioeconomic conditions, since it can generate divergent results.

The divergence in the performance from place to place and from mild TBI to general TBI, turn the comparison and use of the same algorithms in different environments, where the available variables are different, to be difficult [8]. For these reasons, it is still a challenge to find a tool that accurately detects all brain injuries in patients with mild TBI and safely discharges them from hospital, while avoiding unnecessary head CT. This obstacle is due to the fact that the chosen model must have a high sensitivity while keeping the specificity within satisfactory values [57], since sensitivity and specificity are inversely proportional [58].

This way, our study has some limitations. First, the use of the retrospective secondary data can lead to insufficient data and record failures. In addition, the evaluation of data from a single emergency department reflects a restricted scenario. So, these models must be tested and validated in other health centers as well. Considering that occurrence of mild TBI usually is not an isolated event, other variables like sociodemographic and clinical aspects can influence the outcome of the patients with mild TBI, so they should be analyzed to strengthen our study.

Another limitation observed in this study is the low amount of positive imaging results, since the effectiveness of a computational model can be reduced if there is a class imbalance, since most of the time, the majority group tends to overcome the minority, increasing the probability of false-negative results [59].

These limitations show us that although the computational models currently in practice allow researchers to work with easily obtained clinical data, it is important to conduct more robust studies and validate methodology in other health centers. Moreover, future refinements of the algorithm may incorporate other non-invasive measures of traumatic brain injury. For this reason, it is important to develop an integrated and systematic tool to objectively and quantitatively identify patients with mild TBI, with high precision for sensitivity and specificity.

**Fig 4:**
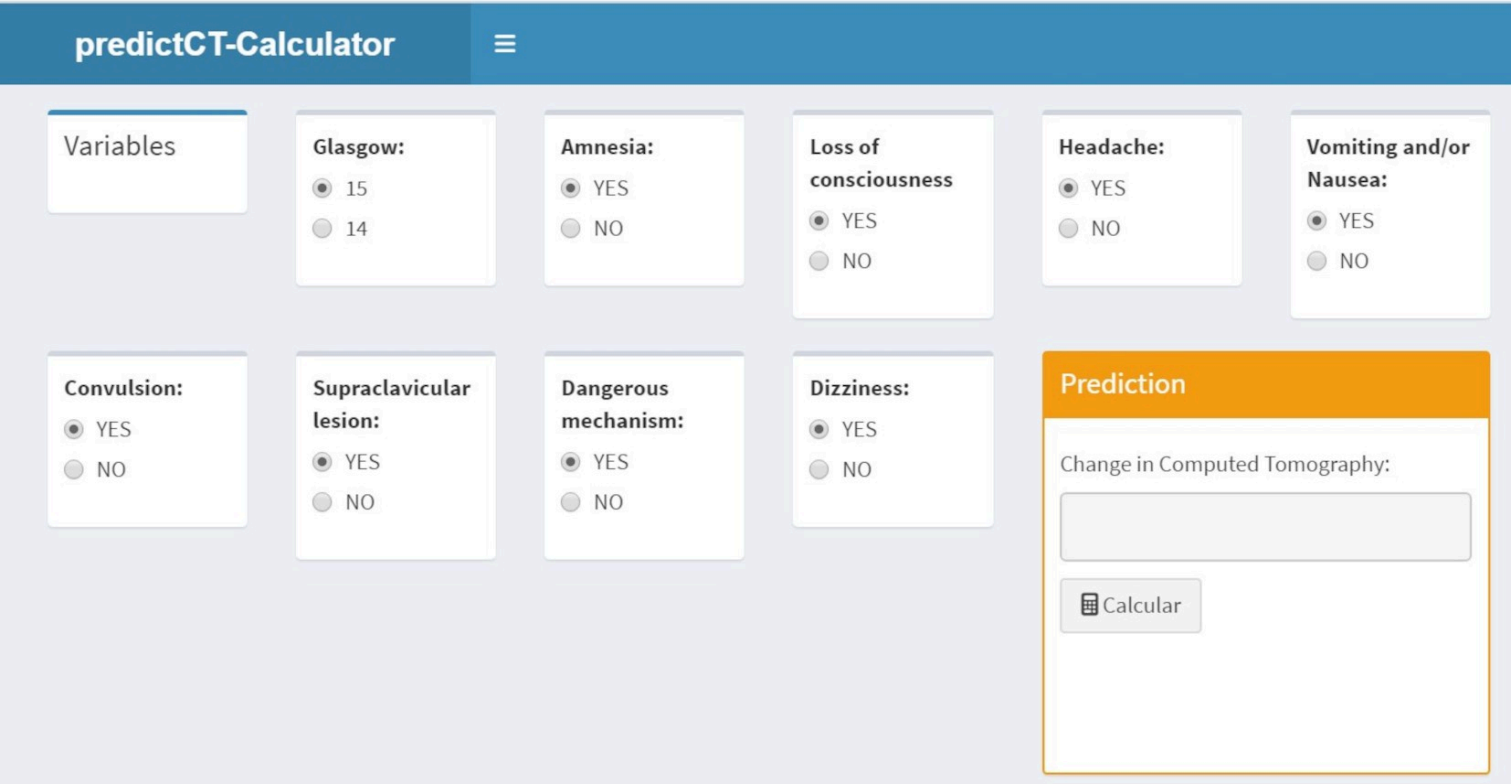
Image of the risk equation prototype.

Moreover, to transform the risk equation into a user-friendly tool that can help physicians to decide whether to perform a brain CT by inserting data in real time during the clinical evaluation of the individuals, a software prototype was developed to calculate the probability of patients having a brain injury (Fig 4).

## Conclusion

Best model (XGB) correctly identified around 70% of all patients with mild TBI, indicating that after the improvement in its performance, this model has a high potential to be used in screening patients with mild TBI. The methodology developed in our study also uses the variables usually collected in Brazilian healthcare services, so it can be used to assist health professionals to manage the financial resources, and improve the quality and safety of patient care in Brazil and other countries with similar conditions, reducing the overcrowding of emergency healthcare services and time to diagnostic of whose show lesions which require neurological intervention in these geographical regions.
