# Correction: Applicability of machine learning technique in the screening of patients with mild traumatic brain injury

**DOI:** 10.1371/journal.pone.0295717

**Published:** 2023-12-06

**Authors:** Miriam Leiko Terabe, Miyoko Massago, Pedro Henrique Iora, Thiago Augusto Hernandes Rocha, João Vitor Perez de Souza, Lily Huo, Mamoru Massago, Dalton Makoto Senda, Elisabete Mitiko Kobayashi, João Ricardo Vissoci, Catherine Ann Staton, Luciano de Andrade

The first author, Miriam Leiko Terabe, is incorrectly noted as the corresponding author. The correct corresponding authors are João Ricardo Vissoci and Catherine Ann Staton. João Ricardo Vissoci’s email address is jnv4@duke.edu and Catherine Ann Staton’s email address is catherine.lynch@duke.edu.
